# Feasibility of Web-Based Single-Session Empowered Relief in Patients With Chronic Pain Taking Methadone or Buprenorphine: Protocol for a Single-Arm Trial

**DOI:** 10.2196/53784

**Published:** 2024-06-06

**Authors:** Morgan R Klein, Beth D Darnall, Dokyoung S You

**Affiliations:** 1 Department of Anesthesiology, Perioperative and Pain Medicine Stanford University School of Medicine Palo Alto, CA United States

**Keywords:** chronic pain, opioid use disorder, methadone, buprenorphine, behavioral medicine, telehealth, psychology

## Abstract

**Background:**

Chronic pain affects tens of millions of US adults and continues to rise in prevalence. Nonpharmacologic behavioral pain treatments are greatly needed and yet are often inaccessible, particularly in settings where medication prescribing is prioritized.

**Objective:**

This study aims to test the feasibility of a live-instructor, web-based 1-session pain relief skills class in an underserved and potentially at-risk population: people with chronic pain prescribed methadone or buprenorphine either solely for pain or for comorbid opioid use disorder (OUD).

**Methods:**

This is a national, prospective, single-arm, uncontrolled feasibility trial. The trial is untethered from medical care; to enhance participants’ willingness to join the study, no medical records or drug-monitoring records are accessed. At least 45 participants will be recruited from outpatient pain clinics and from an existing research database of individuals who have chronic pain and are taking methadone or buprenorphine. Patient-reported measures will be collected at 6 time points (baseline, immediately post treatment, 2 weeks, and months 1-3) via a web-based platform, paper, or phone formats to include individuals with limited internet or computer access and low literacy skills. At baseline, participants complete demographic questions and 13 study measures (Treatment Expectations, Body Pain Map, Medication Use, Pain Catastrophizing Scale [PCS], Patient-Reported Outcomes Measurement Information System [PROMIS] Measures, and Opioid Craving Scale). Immediately post treatment, a treatment satisfaction and acceptability measure is administered on a 0 (very dissatisfied) to 10 (completely satisfied) scale, with 3 of these items being the primary outcome (perceived usefulness, participant satisfaction, and likelihood of using the skills). At each remaining time point, the participants complete all study measures minus treatment expectations and satisfaction. Participants who do not have current OUD will be assessed for historical OUD, with presence of OUD (yes or no), and history of OUD (yes or no) reported separately. Feasibility threshold is set as an overall group treatment satisfaction rating of 8 of 10. In-depth qualitative interviews will be conducted with about 10 participants to obtain additional data on patient perceptions, satisfactions, needs, and wants. To assess preliminary efficacy, we will examine changes in pain catastrophizing, pain intensity, pain bothersomeness, sleep disturbance, pain interference, depression, anxiety, physical function, global impression of change, and opioid craving at 1 month post treatment.

**Results:**

This project opened to enrollment in September 2021 and completed the recruitment in October 2023. The data collection was completed in February 2024. Results are expected to be published in late 2024.

**Conclusions:**

Results from this trial will inform the feasibility and preliminary efficacy of Empowered Relief in this population and will inform the design of a future randomized controlled trial testing web-based Empowered Relief in chronic pain and comorbid OUD.

**Trial Registration:**

ClinicalTrials.gov NCT05057988; https://clinicaltrials.gov/study/NCT05057988

**International Registered Report Identifier (IRRID):**

DERR1-10.2196/53784

## Introduction

### Background

Chronic pain, a condition defined by persistent pain lasting longer than 3 months [1], affects upward of 50 million US adults [2] and is rising in prevalence every year [3]. One-third of individuals with chronic pain report experiencing severe pain [4], about one-third have pain in multiple locations [5], and more than a third have activity-limiting pain [6], indicating a significant toll on life [4]. Indeed, for millions of adults, chronic pain results in reduced productivity, poor quality of life, difficulties with interpersonal relationships, and disability [7,8]. These disturbances are costly to both the individual and the nation, resulting in the loss of billions of dollars each year in health care expenditures and lost productivity [3].

Historically, opioids were often prescribed to treat chronic pain, though prescribing trends have decreased sharply since 2016. For instance, the US national trend analysis of 2014 data revealed that the majority of people taking prescription opioids (79.4%) at that time were taking them long-term [9], a period defined as 90 days or longer. The risk for overdose, misuse, and development of opioid use disorder (OUD) became a growing concern following the declaration of the opioid epidemic as a national public health emergency in 2017 [10]. From 2019 to 2022, the total number of opioid prescriptions decreased by 14.2% (153.6 million in 2019 to 131.8 million in 2022) [11]. Yet, overdose deaths involving prescription opioids remained high, with 8.5 million individuals in the United States reporting opioid misuse in 2022 [12]. While some patients gain durable analgesic benefits from prescription opioids for chronic pain [13], there remains a clear need to provide patients access to the lowest risk pain treatment options.

Nonpharmacologic treatment options have gained substantial clinical attention to optimize pain care at lowest risk. Psychological treatment for chronic pain encompasses a wide variety of interventions focusing on the ability to self-manage pain, pain-related worry, and distress [14]. Multisession cognitive behavioral therapy (CBT), the gold standard psychological therapy for chronic pain [15], has been shown to be effective in reducing pain intensity, pain interference [16], and improving quality of life [17]. Other multisession therapies, such as 8-session acceptance and commitment therapy and 8-session mindfulness-oriented recovery enhancement (MORE), offer slight strategic variations from 8-session CBT and are similarly applied in the management of chronic pain. These pain psychology treatments have shown equivalent effects on pain-related outcomes measures [18] and compatible treatment satisfaction ratings [19,20]. Although MORE was not designed for chronic pain and comorbid OUD, MORE has notably shown effectiveness in reducing opioid craving [21,22]. However, the availability of psychological treatments for chronic pain is limited by a lack of clinicians trained in pain management, time and cost required, and health insurance barriers [3,23]. Furthermore, patients taking medication for OUD (MOUD), including either buprenorphine or methadone, are of specific interest because of their risk for unmanaged pain-triggering relapse. Indeed, patients taking MOUD often have difficulty accessing nonpharmacological treatments and many report unmanaged pain as the primary reason for opioid relapse [24].

Empowered Relief (ER) is a single-session group-based pain relief skills intervention that was developed to reduce treatment burden and increase accessibility to effective low-risk pain care. Results from a randomized controlled 3-arm clinical trial conducted in 263 patients with chronic low back pain revealed that ER was comparable with 8 sessions of CBT [25] in improving pain catastrophizing, pain intensity, pain interference, pain bothersomeness, sleep disturbance, anxiety, depression, and fatigue at 3 months post treatment, with benefits being clinically meaningful. A second randomized controlled trial tested a live-instructor, web-based ER compared with usual care in 105 patients with mixed etiology chronic pain. Results revealed that at 3 months post treatment, ER was superior and significantly reduced pain catastrophizing, pain intensity, pain bothersomeness, anxiety, and sleep disturbance [26]. Digital versions of ER have been used in 2 randomized controlled surgical studies with results showing that it was associated with reduced opioid use after breast cancer surgery [27] and reduced pain up to 3 months after orthopedic trauma surgery [28]. While the collective ER trials are promising, the intervention has not yet been tested as a web-based treatment for people who may have a history of OUD or current OUD, or for people taking medications commonly prescribed for these circumstances. This study is designed to examine whether web-based ER is a plausible and accessible pain treatment option for patients with any chronic pain condition who are receiving methadone or buprenorphine (commonly prescribed medications) for OUD in various clinics in the United States.

### Objectives

This study aims to conduct a single-arm uncontrolled trial to assess the feasibility and preliminary efficacy of a web-based single-session pain education class to participants with chronic pain conditions who are taking methadone or buprenorphine in the United States. We have 3 primary hypotheses. First, we hypothesize that patients with chronic pain who take methadone or buprenorphine will report overall acceptable satisfaction with the brief web-based intervention. Second, we hypothesize that web-based ER will reduce pain metrics (catastrophizing, pain intensity, and pain bothersomeness) as well as sleep disturbance and opioid craving at 1 month post treatment (primary end point) and 3 months post treatment (secondary end point).

## Methods

### Overview and Setting

**Figure 1:**
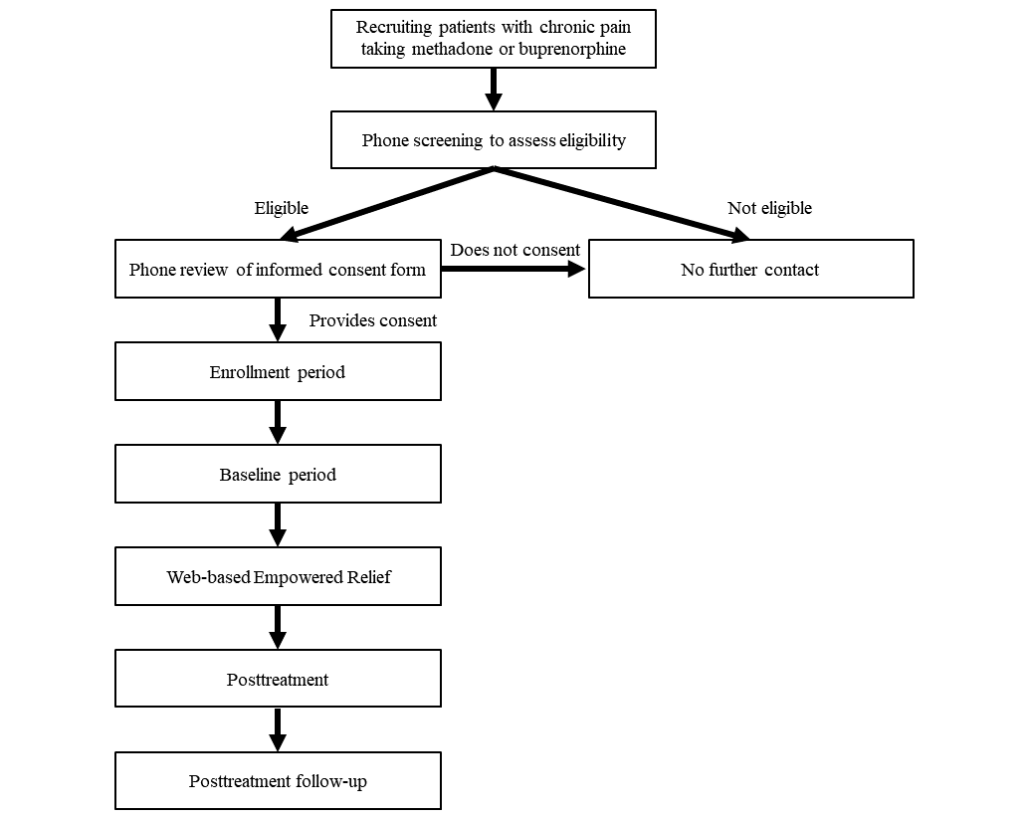
Study design flowchart.

**Figure 2:**
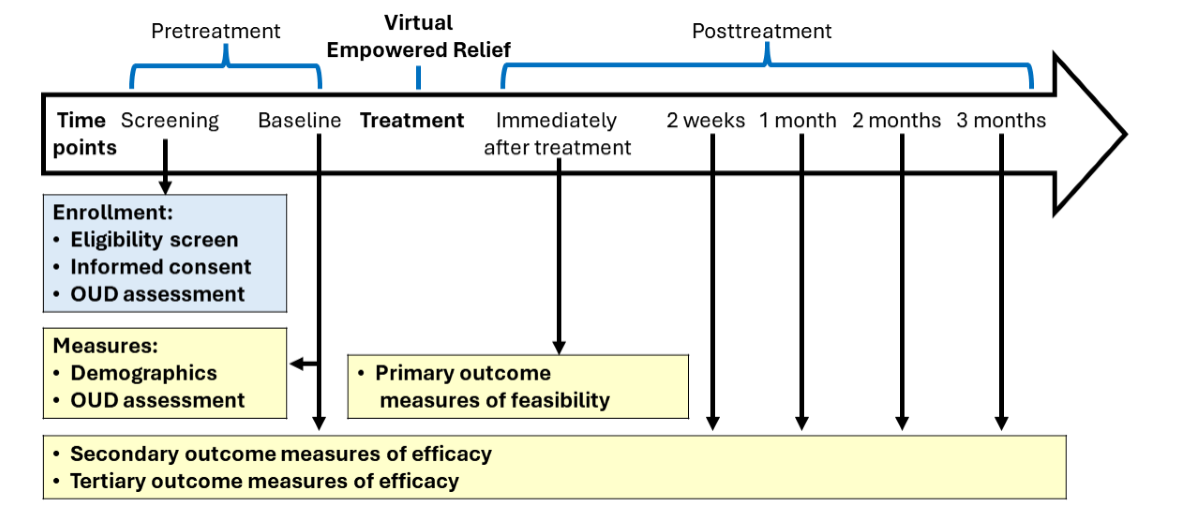
Timeline of enrollment, assessments, and intervention. Note: measures are highlighted in yellow. OUD: opioid use disorder.

We are conducting a single-arm, uncontrolled feasibility clinical trial for patients with chronic pain conditions taking methadone or buprenorphine anywhere in the United States (Figures 1 and 2). All study procedures are performed over the phone or internet (ie, Zoom) and no in-person visits are required. All enrolled participants will attend a web-based ER session. Participants will be assessed for 3 months following the class via web-based surveys. Participants will be surveyed during an initial phone screening; immediately after treatment; 2 weeks; and 1, 2, and 3 months after the class. For clinical outcomes, the primary end point is 1 month (short-term outcomes) and the secondary end point is 3 months (delayed outcomes) post treatment. The primary aim of the study is to report the feasibility of web-based ER using a quantitative treatment satisfaction survey administered immediately post treatment (all participants) and an in-depth qualitative interview with about 10 participants to enhance understanding of patient preferences. The secondary aim is to examine the preliminary efficacy of web-based ER. The secondary outcomes are pain catastrophizing, pain intensity, pain bothersomeness, and sleep disturbance at 1 month and 3 months post treatment. The tertiary outcomes include pain interference, mood, physical function, global impression of change, and cravings at 1 month and 3 months post treatment.

### Ethical Considerations

The protocol for this trial has been approved by Stanford institutional review board (IRB; 60855). This research adheres to the Helsinki Declaration’s ethical standards for research involving human subjects. Informed consent will be obtained from all participants prior to study enrollment. Participants are also notified that they can revoke their consent at any time for any reason without any repercussions. Data access will be exclusively limited to the research team members. After data collection is complete and the results have been published, deidentified data will be preserved. Consequently, any future requests for data access will be limited to this deidentified information, implemented after request approval and conducted in accordance with all IRB regulations. Participants will be compensated up to US $145 in the form of Amazon gift cards ranging from US $10 to US $45 each for successful completion of study surveys with prorated compensation possible. Participants who agreed to participate in the 30-minute qualitative interview will be compensated with an additional US $20 Amazon gift card.

### Data Collection

Data will be collected and managed using REDCap (Research Electronic Data Capture) electronic data capture tools hosted at Stanford University [29,30]. REDCap is a secure, HIPAA (Health Insurance Portability and Accountability Act) compliant, web-based software platform designed to support data capture for research studies, providing (1) an intuitive interface for validated data capture, (2) audit trails for tracking data manipulation and export procedures, (3) automated export procedures for seamless data downloads to common statistical packages, and (4) procedures for data integration and interoperability with external sources. All study measures will be completed using a web-based survey platform unless a participant requests paper-based or telephonic format. All web-based surveys will be completed directly by the participants and sent by email through a secure, participant-specific link.

### Study Sample and Recruitment

Participants will be recruited nationally through pain and addiction clinics by patient referral from physicians and clinic staff briefed on the treatment research opportunity. This study invites patients with any chronic pain conditions who are also taking methadone or buprenorphine; we will recruit at least 45 participants completing study measures at the primary end point. Interested individuals will be scheduled to complete a brief phone screening interview with study staff to assess eligibility. Individuals who meet the eligibility criteria will be contacted by phone to complete informed consent and study enrollment. Enrolled participants will complete an assessment of OUD symptoms at this time.

### Inclusion and Exclusion Criteria

Eligibility criteria include ongoing body pain for more than half of the days for ≥3 months, taking either methadone or buprenorphine, 18 years of age or older, having access to a device with video call capabilities, and verbal English fluency (written English fluency is not required because participants may choose 1 of 3 survey options: web-based, paper, or research coordinator–assisted phone surveys). Furthermore, internet or computer access is not required if referring clinics can accommodate the Zoom (Zoom Technologies Inc) ER group meeting for 2 hours. Individuals who are pregnant or have previously received ER will be excluded for this feasibility study to minimize the incompletion of research tasks during the 3-month study period and maintain the homogeneity of the sample.

### One-Session ER Intervention

ER was developed in 2013 at the Stanford University Division of Pain Medicine with clinician certification workshops initiating at Stanford University in 2019 [29,31]. ER provides an accessible, low-risk ntervention for pain management. The didactic 2-hour class may be delivered to large groups of patients. A certified instructor uses a structured PowerPoint slide deck and instructor manual to deliver content that includes pain neuroscience education, experiential and interactive exercises, 3 core pain management skills, and a guided binaural relaxation audio application that participants download onto their smartphone or other device. Instructors assist participants with the creation of a personalized plan that uses the skills presented in the course and can be implemented outside of the class. ER has been shown as multidimensional benefits to participants with chronic pain conditions with both short-term and long-term effects.

For this study, the ER class will be offered once monthly to small groups that consist of 5-20 participants each. The class is hosted through the Zoom platform. To ensure participant confidentiality, participants’ cameras are off and they are asked to display only first names. Furthermore, the classes are not recorded. Participants can receive the study materials via email or mail and can receive the guided binaural relaxation audio file by direct app download or by email.

### Safety Monitoring and Adverse Experiences

Prior research has documented no ER study–related adverse events [25,26]. For this study, participants are asked to report any adverse events or new problems they have experienced since their last survey at each survey time point. The adverse event forms are reviewed by the study staff and a determination is made regarding event severity and whether the event is related to the study or not. Any reported adverse event cases will be discussed in team meetings and reported to the IRB annually. All serious adverse event cases will be evaluated by the study staff and principal investigator (DY) within 24 hours of acknowledgment of the incident and immediately reported to the Stanford IRB.

### Study Measures

#### Measures

We collect clinical and demographic information listed in Table 1. The 11 *DSM-5* OUD [32] symptoms are assessed to evaluate both present and lifetime OUD status. Diagnostic criteria for OUD are met when 2 or more symptoms are endorsed. The 6-item Stanford Expectation of Treatment Scale [33] is administered to assess positive and negative expectation of upcoming treatment on an 11-point Likert scale (α=.86). We will calculate the means of negative and positive treatment expectations, which will be compared with previous in-person ER class (mean values 2.29 with SD of 1.34 for negative expectation and 3.71 with SD of 1.30 for the positive expectation [25]). The Body Map [34], a whole body topography with 36 anterior and 38 posterior segments, is administered to assess pain locations (1-week test-retest reliability=0.93). The 13-item Pain Catastrophizing Scale (PCS) [35] is administered to assess the levels of pain catastrophizing (α=.87) on a 5-point scale from 0 (not at all) to 4 (all the time). The PCS measures a multifaceted construct with 3 subscales: rumination, magnification, and helplessness about pain. The PCS total score ranges from 0 to 52, with higher scores indicating higher levels of pain catastrophizing. Pain bothersomeness [36,37] is assessed with a single item on a scale of 0 (not at all bothersome) to 10 (extremely bothersome) in the past 7 days. This single-item measure of pain bothersomeness has shown to be predictive of poor functional outcomes in the future [38] and be sensitive to intervention-related changes [25]. The Patient-Reported Outcomes Measurement Information System (PROMIS)–Pain Intensity item [39] is used to assess average pain in the past 7 days on a 0-10 scale. In addition, short-form versions of the PROMIS [40] measures of pain interference, physical function, fatigue, sleep disturbance, depression, anxiety, and social isolation are administered to assess the previously observed outcomes of in-person ER intervention [25]. The PROMIS measures are reported on a T-score metric (mean 50T, SD 10), with higher scores indicating more symptoms or greater function of the domain measured. The Patient Global Impression of Change [41,42] is a 6-item measure to assess perceived progress after the ER intervention. The Patient Global Impression of Change is rated on a 1 (very much worse) to 7 (very much improved) scale. Next, the level of craving for opioid medication is assessed with a single item (“how much do you crave opioid medication now?”) rated on a scale of 0 (not at all) to 10 (extremely) as others have demonstrated opioid-craving reductions in patients with chronic pain and OUD following receipt of pain management programs [43-45]. Feasibility is assessed using 3 items (perceived usefulness, participant satisfaction, and likelihood of using the skills) from a treatment satisfaction and acceptability measure that includes 11 questions (Table 2) about the class content and format (eg, overall satisfaction, understandability, relevancy, worry related to the web-based class, and length of the class) [46] on a 0-10 scale. Finally, participants are asked to report any adverse events and major life events during the study period in a text format.

**Table 1:** Study measures and time point of administration.

Measure	Description	Enrollment	Baseline	Posttreatment	Follow-up^a^ (~3 months)
*DSM-5*^b^ OUD^c^ [32]	Assessing opioid use disorder symptoms in lifetime and the past 12 months (11 items)	✓	✓		
Demographics	Gender, age, ethnicity, race, education, employment, marital status, household income, housing status, and self-reported medical diagnoses		✓		
Medication use	Self-reported name, start date, dose, and frequency of current medications		✓		✓
Stanford Expectation of Treatment Scale [33]	Expectations and concerns about the treatment on a 7-point scale (6 items)		✓		
Body Map [34]	A body diagram to indicate location of pain		✓		✓
Pain Catastrophizing Scale [35]	Thoughts and emotions when experiencing pain on a scale ranging from 0 to 4 (13 items)		✓		✓
Pain Bothersomeness [36,38]	Assessing how “bothersome” the pain is on a 0-10 scale (1 item)		✓		✓
PROMIS^d^ Measures [39,40]	Pain intensity, pain interference, physical function, fatigue, sleep disturbance, depression, anxiety, and social isolation		✓		✓
Opioid Craving Scale [47]	Assessing the strength of opioid cravings on a 0-10 scale (1 item)		✓		✓
Patient Global Impression of Change [41]	Perceived changes in social, recreational, and occupational activities following the intervention measured on a 7-point scale (6 items)		✓		✓
Class Satisfaction and Acceptability [26]	Experience with ER^e^ class including overall satisfaction, relevancy and usefulness of material, and ease of using Zoom platform (11 items)			✓	
Treatment Change and Adverse Events	Any major life events, new injuries/illnesses, and changes in lifestyle and treatment				✓

^a^Two weeks and 1, 2, and 3 months after treatment.

^b^DSM-5: Diagnostic and Statistical Manual of Mental Disorders (Fifth Edition)

^c^OUD: Opioid Use Disorder.

^d^PROMIS: Patient-Reported Outcomes Measurement Information System.

^e^ER: Empowered Relief.

**Table 2:** Eleven-item class satisfaction and acceptability measure^a^.

Questions	Scale
1. Was the content easy to understand?	0 (not understandable at all) to 10 (completely understandable)
2. How relevant was the class to you?	0 (not relevant at all) to 10 (completely relevant)
3. *How useful was the information presented in the class?*	0 (completed useless) to 10 (very useful)
4. *Please rate your overall satisfaction with the class.*	0 (very dissatisfied) to 10 (completely satisfied)
5. *How likely are you to use the skills and information you learned?*	0 (not at all likely) to 10 (highly likely)
6. Rate your likelihood to recommend this class to another person who has chronic pain.	0 (would not recommend) to 10 (absolutely would recommend)
Please indicate how much you agree with the following statements: 7. It was easy for me to operate Zoom as a platform for attending the class. 8. While attending the Zoom class, I worried about privacy. 9. I felt comfortable engaging with the Zoom instructor and class participants. 10. I felt connected to the instructor.	0 (strongly disagree) to 10 (strongly agree)
11. Would you prefer to have a single class (2 hours) or two classes (1 hour each)?	Prefer single class (2 hours) Prefer two classes (1 hour each) Other (if other, explain)

^a^Text in italics indicates primary outcomes.

#### Baseline (Pretreatment) Assessment

Following enrollment, participants will be asked to provide demographic information and complete all baseline study measures during a 2-week window prior to attending the web-based ER intervention. The *DSM-5* OUD symptoms are assessed by a trained study staff at enrollment and with a self-report survey at baseline that assesses current and lifetime history of OUD.

#### Posttreatment

Immediately following receipt of ER, participants complete the treatment satisfaction survey. Participants complete surveys at the following 4 posttreatment time points: 2 weeks, 1 month (primary end point), 2 months, and 3 months (secondary end point). Posttreatment surveys will assess the benefits of the ER class over time.

#### Primary Outcome Measures of Feasibility

Feasibility will be evaluated in qualitative and quantitative methods (treatment satisfaction survey). Of the 11 feasibility questions, the 3 primary outcomes are overall treatment satisfaction [26], perceived usefulness, and likelihood of using the skills learned. We will also conduct about 10 qualitative interviews with open-ended questions (Textbox 1) to understand participants’ perception of the study and to request feedback on study design including the use of Zoom platform, recruitment method, survey burden, and compensation.

**Ten-item qualitative interview.**

1. Please tell us about the content of the class. Were any parts of the class difficult to understand?
2. Did any parts of the class make you feel uncomfortable?
3. Is there anything we can do differently for the next participants?
4. Did you have any issues attending the Zoom class?
5. Did you have any concerns about our recruitment methods? As a reminder, we posted a flyer, we asked your health care provider at the clinic to introduce our study, and we reached out to you for the phone screening.
6. Do you have any suggestions about how we can better advertise our pain study to people who may benefit from our study?
7. Did you have any difficulties in completing the web-based survey, via phone, or on a paper? (If yes) Tell us your difficulties in completing the web-based survey, via phone, or on a paper.
8. Can we do anything differently to help people complete the survey?
9. What do you think about the study compensation?
10. Did you have any concerns while you participated in this study?

#### Secondary Outcome Measures of Efficacy

The secondary outcome measures are pain catastrophizing [35], pain intensity [39], pain bothersomeness [38], and sleep disturbance [40] as reported by the patient at the 1-month posttreatment time frame. We will also evaluate the long-term outcomes at 2 and 3 months post treatment.

#### Tertiary Outcome Measures of Efficacy

Other outcome measures include pain interference [48], physical function [40], fatigue [40,49], depression [40], anxiety [40], social isolation [40], the global impression of change [41,42,50], and the opioid-craving levels [47]. These outcomes will all be assessed at the 1-, 2-, and 3-month posttreatment time points.

### Statistical Analyses

#### Sample Size Calculation

In a previous single-arm ER pilot study involving mixed etiology chronic pain, a sample size of 57 participants was needed to detect a significant time effect on pain catastrophizing scores at 1 month post treatment (Cohen *d*=1.15, large effect) [46]. In addition, when conducting a repeated-measures ANOVA with a prior in-person randomized controlled trial (RCT) ER class data only [25], a sample size of 52 was needed to detect a significant time effect. The analysis also revealed moderate to large effects in reducing pain catastrophizing (Cohen *d*=0.91), pain rating (Cohen *d*=0.45), pain bothersomeness (Cohen *d*=0.84), and sleep disturbance scores (Cohen *d*=0.47) at 1 month post treatment. To calculate sample sizes for this study, power analysis was conducted using the G*Power 3.1.9.7. Specifically, we calculated sample sizes needed to detect a moderate effect (ƒ=0.25) for a 1-way repeated-measures ANOVA, with α level of .05, 3 data points, and 0.50 correlation coefficient among repeated measures. The result indicated that the sample sizes of 36, 43, and 59 would yield the power of 90%, 95%, and 99%, respectively. Originally, 20% attrition rate was noted in the in-person ER class [25] and this pilot study initially estimated high attrition rate (ie, 50%). However, the observed attrition rate of this ongoing pilot study has been 25%, so we will recruit at least 45 participants to detect at least moderate effect sizes with a power of 90% and α level of .05.

#### Primary Outcome Analysis to Determine Feasibility

To determine the feasibility of a future RCT, we will evaluate the following criteria.

1. At least 75% of enrolled participants will attend the web-based ER class [27].
2. Demographic data (sex, age, race or ethnicity, education, income) will be compared between those who drop out and complete the study at the primary end point.
3. The mean scores of the 3 primary feasibility outcome measures will meet or exceed an 80% threshold of acceptability (8 or higher on a 0-10 scale) [28].
4. The qualitative interview data will be analyzed to evaluate perceived feasibility and to identify areas for improvement in the study design for a future, large-scale RCT [51].

#### Secondary and Tertiary Outcome Analysis to Examine the Preliminary Efficacy

To test the secondary and tertiary hypotheses, repeated-measures ANOVAs will be conducted to examine whether the secondary and tertiary outcomes will be significantly changed over time. False discovery rate adjustments will be used to control for type 1 error for the multiple comparisons. If the ANOVA results reveal significant change in the outcomes over time, post hoc analyses will be conducted to examine whether the secondary and tertiary outcomes will be improved by 1 month (primary end point) or 3 months (secondary end point) after the web-based ER class.

## Results

An IRB approved all the study procedures on August 31, 2021. This project opened to enrollment in September 2021. Recruitment began in May 2022 and ended in October 2023. The end date for data collection was February 2024. Findings will be published upon completion of the study. The results are expected to be published by the end of 2024.

## Discussion

We anticipate that the internet-delivered ER will be a feasible treatment for patients with chronic pain taking methadone or buprenorphine. In addition, our findings will support the primary and secondary hypotheses. Specifically, the internet-delivered ER will be a satisfactory and potentially efficacious treatment in this patient population as evidenced by the mean rating of the treatment satisfaction of ≥8 of 10 and significant improvement in pain-related outcomes, sleep disturbance, and opioid craving at 1 month (primary end point) and 3 months later (secondary end point).

Through this trial, we will determine whether the web-based 1-session ER intervention is feasible and effective for individuals with chronic pain who are taking methadone or buprenorphine. Qualitative data will yield richer information on participant perceptions of the intervention, the group-based and web-based format, and study procedures. Combined, quantitative and qualitative data will determine whether a fully powered RCT is warranted and whether refinements are needed. If study results suggest that the web-based ER has acceptable feasibility and shows preliminary efficacy, our next step would be to conduct a larger RCT. If this study reveals that the web-based ER does not significantly improve any of the primary and secondary outcomes, we will refine the protocol based on participants’ feedback and consider next steps in this line of research (but not a next-step national RCT). Recognizing that a web-based intervention or clinical study would benefit from being fully embedded into the clinical environment, this study is a first step in that direction. Theoretically, on the one hand, this study benefits from being untethered to medical care in that participants may feel safer disclosing sensitive information about opioid use, craving, or substance use. On the other hand, the study is disadvantaged by being disconnected from clinics in that there is no in-house staff to promote the study or deliver the study treatment; rather, the study relies on colleague clinicians sharing information with their patients about an outside and unaffiliated study.

The single-session, 2-hour web-based ER is a low-burden, easily accessible, and scalable pain management intervention compared with traditional treatments involving 8-12 sessions [52,53]. A prior RCT conducted in people with chronic low back pain demonstrated that it was noninferior to 8 weeks of CBT (16 hours) for reducing pain catastrophizing, pain intensity, pain interference, sleep disturbance, and pain bothersomeness, and other outcomes at 3 months post treatment [25]. A web-based ER was also shown to have clinically meaningful efficacy in reducing a range of symptoms at 3 months post treatment for people with mixed etiology chronic pain [26]. Building on these studies, we have designed this web-based ER national study to reach people with chronic pain who are receiving medications that are often prescribed for pain and OUD (current or historical) and who likely have poor access to pain psychology treatment. To facilitate the study of web-based treatments for this underserved patient population, we provide a detailed research protocol, with a goal of sharing this resource widely with clinicians and researchers who work with this patient group. Limitations of this study include the single-arm design, reliance on self-report data, an enrollment criterion for medications commonly used to treat pain and OUD, and reliance on clinician colleague referrals. Despite these limitations, this study may yield important information. If the single-session web-based ER intervention is shown to be feasible and have preliminary efficacy, a future rigorously designed multisite or national RCT would be warranted, one that includes longer-term follow-up and objective measures alongside the participant self-report data (eg, medical records data). This line of research may provide a viable and accessible pain care option for people living with any type of chronic pain and past or current OUD. Future research may also explore whether the intervention favorably impacts MOUD adherence and relapse prevention.

## Abbreviations

CBT: cognitive behavioral therapy
ER: Empowered Relief
HIPPA: Health Insurance Portability and Accountability Act
IRB: institutional review board
MORE: mindfulness-oriented recovery enhancement
MOUD: medication for opioid use disorder
OUD: opioid use disorder
PCS: Pain Catastrophizing Scale
PROMIS: Patient-Reported Outcomes Measurement Information System
RCT: randomized controlled trial
REDCap: Research Electronic Data Capture

## Data Availability

The data sets generated and analyzed during this study are available upon study team approval of written requests with analytic plan included in the request.
